# STRIDER (Sildenafil TheRapy in dismal prognosis early onset fetal growth restriction): an international consortium of randomised placebo-controlled trials

**DOI:** 10.1186/s12884-017-1594-z

**Published:** 2017-12-28

**Authors:** A. Pels, L. C. Kenny, Z. Alfirevic, P. N. Baker, Peter von Dadelszen, C. Gluud, C. T. Kariya, B. W. Mol, A. T. Papageorghiou, A. G. van Wassenaer-Leemhuis, W. Ganzevoort, K. M. Groom, the international STRIDER Consortium

**Affiliations:** 1 Academisch Medisch Centrum, Meibergdreef 9, 1105 AZ Amsterdam, The Netherlands; 2 University College Cork, Cork, Ireland; 3 University of Liverpool, Liverpool, UK; 4 College of Life Sciences, University of Leicester, Leicester, UK; 5 Department of Women’s and Children’s Health, School of Life Course Sciences, King’s College London, London, UK; 6 Copenhagen Trial Unit, Centre for Clinical Intervention Research, Rigshospitalet, Copenhagen University Hospital, Copenhagen, Denmark; 7 The Robinson Research Institute, School of Paediatrics and Reproductive Health, University of Adelaide, Adelaide, SA 5000 Australia; 8 University of Oxford, Oxford, UK; 9 University of Auckland, Auckland, New Zealand

**Keywords:** Fetal growth restriction, Placental insufficiency, Sildenafil, Randomised placebo controlled trial, Neonatal mortality, Neonatal morbidity

## Abstract

**Background:**

Severe, early-onset fetal growth restriction due to placental insufficiency is associated with a high risk of perinatal mortality and morbidity with long-lasting sequelae. Placental insufficiency is the result of abnormal formation and function of the placenta with inadequate remodelling of the maternal spiral arteries. There is currently no effective therapy available. Some evidence suggests sildenafil citrate may improve uteroplacental blood flow, fetal growth, and meaningful infant outcomes. The objective of the Sildenafil TheRapy In Dismal prognosis Early onset fetal growth Restriction (STRIDER) collaboration is to evaluate the effectiveness of sildenafil versus placebo in achieving healthy perinatal survival through the conduct of randomised clinical trials and systematic review including individual patient data meta-analysis.

**Methods:**

Five national/bi-national multicentre randomised placebo-controlled trials have been launched. Women with a singleton pregnancy between 18 and 30 weeks with severe fetal growth restriction of likely placental origin, and where the likelihood of perinatal death/severe morbidity is estimated to be significant are included. Participants will receive either sildenafil 25 mg or matching placebo tablets orally three times daily from recruitment to 32 weeks gestation.

**Discussion:**

The STRIDER trials were conceived and designed through international collaboration. Although the individual trials have different primary outcomes for reasons of sample size and feasibility, all trials will collect a standard set of outcomes including survival without severe neonatal morbidity at time of hospital discharge. This is a summary of all the STRIDER trial protocols and provides an example of a prospectively planned international clinical research collaboration. All five individual trials will contribute to a pre-planned systematic review of the topic including individual patient data meta-analysis.

**Trial registrations:**

New Zealand and Australia: ACTRN12612000584831. Registered 30/05/2012.

Canada: NCT02442492. Registered 05/05/2015.

Ireland: CT 900/572/1. Registered 15/07/2015.

The Netherlands: NCT02277132. Registered 29/09/2014.

United Kingdom: ISRCTN39133303. Registered 31/07/2014.

## Background

An estimated 0.4% of pregnancies worldwide are complicated by severe early-onset (<28 weeks gestation) fetal growth restriction (FGR) caused by placental insufficiency. This patient group utilises disproportionate amounts of obstetric care and has a high likelihood of premature birth, both for fetal and for secondary maternal indications such as the development of the maternal syndrome of pre-eclampsia. As these growth-restricted infants are usually born very preterm, they carry additional significant risks of major and minor neonatal morbidity, and long-term health sequelae if they survive. These risks are not only related to gestational age at birth, but also to the degree of FGR. Survival proportions for severely growth-restricted fetuses very remote from term (<28 weeks’ gestation) vary between 7 and 33% [1–3] and less than one third of these fetuses will survive their neonatal intensive care unit (NICU) stay without significant neurodevelopmental sequelae [4].

The diagnosis of early onset FGR is often missed but even when diagnosed based on growth parameters below the normal range (<10th, <5th, or <3rd centile) with or without evidence of abnormal fetal and maternal Doppler waveforms there are currently no specific evidence-based therapies available. Non-specific interventions may include lifestyle modifications such as reducing or stopping work, stopping aerobic exercise, rest at home, and hospital admission for rest and surveillance. These interventions are used in the belief that rest will reduce the steal from the utero-placental circulation to the glutei and quadriceps muscles but are not based on any good quality evidence. In the absence of proven therapeutic interventions, standard clinical management consists of counselling, intensive monitoring, and timely delivery once a fetus has reached a viable gestation and size but often results in extreme preterm birth.

Doppler waveform analysis of pregnancies complicated by severe FGR suggests compromised utero-placental circulation and placental hypo-perfusion [5, 6]. Sildenafil, a phosphodiesterase inhibitor, potentiates the action of nitric oxide thus causing vasodilatation [7]. It is therefore possible that sildenafil may affect utero-placental circulation and perfusion resulting in improved gaseous and nutrient exchange and improved fetal growth and well-being. Animal and preclinical studies support this concept [8–13].

Although the number of women using sildenafil in pregnancy is low, it is increasingly used in pregnancy for maternal cardiac indications with no reports of adverse maternal or fetal effects [14–21]. In a small randomised clinical trial in women with early onset pre-eclampsia, sildenafil use had no demonstrable effect on prolongation of pregnancy, but provided further reassurance on drug safety profile in pregnancy [16]. Sildenafil has been used in a small cohort of women in the setting of early onset FGR. In an observational study by STRIDER collaborators in Vancouver, Canada, there was a tendency towards more live-born children with survival intact to primary discharge for women treated with sildenafil when compared to women with pregnancies at similar risk but not receiving sildenafil [17]. From the limited observations to date there are no concerns of adverse maternal, fetal, neonatal, or infant effects associated with sildenafil use in pregnancy [14–21].

On the basis of this preliminary research, some centres have already adopted treatment with sildenafil [18, 19]. However, there is no clear evidence of true health benefits and, more significantly, potential harm has not yet been excluded. Use of sildenafil for other indications in clinical trials and in post-market reporting has highlighted adverse drug reactions including headache, flushing, nasal congestion, and impaired vision and, rare, serious consequences such as myocardial infarction, arrhythmias, and stroke [7]. More specific to pregnancy and FGR, sildenafil’s vasodilatory properties may cause a transient decrease in blood pressure with potential to adversely affect the most at risk fetuses (those with absent or reversed end diastolic flow on Doppler waveform analysis), via a reduction in critical utero-placental flow or by a direct effect on fetal vasculature. Prolonging pregnancy in FGR has the potential to shift the survival curve but will not necessarily have the same positive impact on short term outcomes and long term well-being. Well-designed, appropriately powered randomised placebo-controlled trials are required before implementation into clinical practice should be considered.

The overarching hypothesis of this collaboration of clinical trials is that sildenafil citrate compared with placebo will improve fetal growth and wellbeing, allowing prolongation of pregnancy leading to a decrease in the rate of fetal and neonatal mortality and severe morbidity.

## Methods/design

### Design of the trials

Randomised placebo-controlled trials in New Zealand/Australia, Canada, Ireland, the Netherlands, and the United Kingdom including participants during 2014 to 2020. Trials are independently funded and executed and will be independently reported but all will contribute to a prospectively planned systematic review including individual patient data (IPD) meta-analysis [22]. Each trial is prospectively registered New Zealand and Australia: ACTRN12612000584831, Canada: NCT02442492, Ireland: CT 900/572/1, the Netherlands: NCT02277132 and the United Kingdom: ISRCTN39133303. Study protocols can be freely accessed URL [23–27].

### Setting

Clinical trials are taking place in tertiary care centres in New Zealand/Australia, Canada, Ireland, the Netherlands, and the United Kingdom. A single trial management service hosted at the University of British Columbia, Canada, is being used by each trial and provides a randomisation service and electronic data collection system. IPD meta-analysis and systematic review will be performed by a trial and systematic review service unit in Denmark, The Copenhagen Trial Unit.

### Participants

Pregnant women referred to tertiary care referral centres for evaluation and management of severe early-onset FGR at gestational ages <30 weeks.

### Inclusion and exclusion criteria

Individual trial inclusion and exclusion criteria are shown in Table 1. The differing inclusion criteria between individual trials allow for variations in feasibility, local standards, and investigator choice.

**Table 1:** Trial characteristics of each STRIDER trial

	Australia/ New Zealand	Canada	Ireland	The Netherlands	United Kingdom
Number of participants	122	90	112	354	112
Number of recruiting sites	12	10	6	10	18
GA (weeks)	22^+0^–29^+6^	18^+0^–27^+6^	22^+0^–29^+6^	20^+0^–29^+6^	22^+0^–29^+6^
Inclusion criteria	• Singleton pregnancy • Expectant management • 22^+0^–27^+6^ weeks: AC ≤3rd %ile • 28^+0^–29^+6^weeks: EFW <700 g.	• Singleton pregnancy • Expectant management • AC <10th %ile AND/OR • Reduced fetal growth (AC interval < 50% of expected) with either prior severe early onset FGR with adverse perinatal outcome OR abnormal uterine artery waveform in index pregnancy; OR EFW <700 g) • Serum PlGF levels < 5th %tile for GA	• Singleton pregnancy • Expectant management • EFW OR AC <10th centile • absent or reversed EDF in umbilical artery	• Singleton pregnancy • Expectant management • 20^+0^–27^+6^wk: AC <3rd %ile OR EFW <5th %ile • 28^+0^–29^+6^wk: EFW <700 g • Likely placental origin defined by a. uterine artery notching OR b. abnormal flow of umbilical artery or middle cerebral artery OR c. maternal hypertensive disorder OR d. low PlGF in point-of-care assessment	• Singleton pregnancy • Expectant management • EFW OR AC <10th centile • absent or reversed EDF in umbilical artery
Exclusion criteria	• Known major fetal anomaly/syndrome/congenital infection deemed as likely cause for FGR • Known fetal aneuploidy • Any contra-indication to sildenafil use	• Maternal age ≤ 18 years • Known fetal aneuploidy, fetal anomaly/syndrome/congenital infection • Any contra-indication to sildenafil use • Cocaine/ crystal meth use • Pre-eclampsia or gestational hypertension • HIV positive/ maternal heart disease • Receiving Prazosin, peripheral alpha-blockers, nitrates, vasoconstrictors	• Maternal age ≤ 18 years • Known or suspected structural or chromosomal fetal abnormality • Any contra-indication to sildenafil use • Cocaine use	• Maternal age ≤ 18 years • Known congenital malformation or infection • Any contra-indication to sildenafil use • Cocaine use • Use of cyp3A5 inhibitor	• Maternal age ≤ 16 years • Known or suspected structural or chromosomal fetal abnormality • Any contra-indication to sildenafil use • Cocaine use
Treatment period	Until delivery, demise or 31^+6^ weeks of gestation, whichever comes first	Until delivery, or 31^+6^ weeks of gestation, whichever comes first	Until delivery, or 31^+6^ weeks of gestation, whichever comes first	Until delivery, or 31^+6^ weeks of gestation, whichever comes first	Until delivery, or 31^+6^ weeks of gestation, whichever comes first
Stratification criteria	i. Umbilical artery EDF ii. GA range < 24 weeks vs ≥ 24 weeks	i. Centre	i. Centre ii. GA range (22^+0^–25^+6^) vs (26^+0^–29^+6^)	i. Centre	i. Centre ii. GA range (22^+0^–25^+6^) vs (26^+0^–29^+6^)
Primary outcome	Fetal growth velocity determined by AC growth velocity	^a^GA at delivery	Prolongation of pregnancy for 1 week as a surrogate for long term morbidity	Intact perinatal survival to term age without evidence of either severe CNS injury or non-CNS severe morbidity	Prolongation of pregnancy for 1 week as a surrogate for long term morbidity
Randomisation	^b^Centralised, computer based				
Blinding	Specifically manufactured visually matching active drug and placebo	Visually matching active drug and placebo by over encapsulation	Visually matching active drug and placebo by over encapsulation	Specifically manufactured visually matching active drug and placebo	Visually matching active drug and placebo by over encapsulation
Data mangement	^b^Centralised, computer based				

*GA* Gestational age, *EFW* Estimated Fetal Weight, *AC* Abdominal Circumference, *PlGF* Placental Growth Factor, *EDF* End Diastolic Flow, *HIV* Human Immunodeficiency Virus, *CNS* Central Nervous System

^a^The first planned primary outcome for the Canadian STRIDER trial was fetal growth velocity [22]. This was changed to an increase in gestational age at delivery following a funding review and assessment of feasibility

^b^Randomisation services and electronic data collection and management service (RedCap) provided to each individual trial by single co-ordinating centre at University of British Columbia, Canada

### Ethics and informed consent

All trials have obtained appropriate local ethics approvals. All participating women are provided with written and verbal information regarding the trial they are going to enter and provide signed informed consent in advance of participation. Any protocol modifications once the trials are underway will be reviewed by the local Ethics Committee. Appropriate clinical trial insurance is in place for participants of each trial in the event that any participants experiences harm as a consequence of participation in these trials.

### Randomisation

*R* programming statistical software was used for generating the allocation lists. The R package *blockrand* function was modified to allow for varying numbers of strata. For each trial an allocation list was generated for each level of stratification variable. Each allocation list had a length of sample size +20%*.* Block sizes used are specific to each individual trial.

### Experimental and control interventions

Participants will receive oral sildenafil 25 mg or matching placebo tablets (table 1) three times daily from randomisation until delivery, fetal demise, or 32 weeks gestation (whichever occurs first). Participants, researchers, dispensing pharmacists, clinicians and outcome assessors will remain unaware of treatment allocation for duration of trial. All other interventions will be provided according to local practice. Treatment code may be revealed in event of a serious adverse event where the responsible clinician deems this information to be crucial to provide on-going safe clinical care.

### Sample size estimation

Assumptions for sample size estimations were made on the basis of clinical relevance, local audit, and a pilot cohort [17]. Investigators for each individual trial made their own estimations, based on the choice of primary outcome and the following variables:

The New Zealand/Australian STRIDER trial has a primary outcome of fetal growth velocity determined by abdominal circumference (AC) growth velocity. Using data from the pilot cohort [17] to estimate a difference of 50% in placebo-treated versus 80% in sildenafil-treated of pregnancies with an increased post-randomisation AC growth velocity, 58 women will be randomised per group, (two-sided α of 0.05 and 90% power to detect this difference). Allowing for a 5% drop-out rate, the total sample size will be 122 women.

Three of the STRIDER trials have a primary outcome relating to the interval between randomisation and birth. In the UK STRIDER and Irish STRIDER trials, 1 week (7 day) difference in mean randomisation and birth interval is considered to be clinically important. Internal audits of early-onset FGR cohorts revealed an average diagnosis-delivery interval of 20 days with standard deviation of 11 days. In order to confirm or refute that sildenafil can prolong pregnancy by 1 week compared with placebo, 52 women will be randomised per group in each trial (two-sided α of 0.05 and 90% power to detect this difference). Allowing for a 5% drop-out rate, the total sample size will be 112 women in each trial. Based on local pilot cohort experience, the Canadian STRIDER trial assumes a 16-day difference in mean gestational age at delivery, 189 days (placebo-treated) and 205 days (sildenafil-treated) [17]. 41 women will be randomised per group (two-sided α of 0.05 and 80% power to detect this difference). Allowing for a 10% drop-out rate, the total sample size will be 90 women.

The Dutch STRIDER trial has a primary outcome of intact infant survival until hospital discharge. Assuming a 29% (placebo-treated) and 44% (sildenafil-treated) proportion of intact infant survival until hospital discharge, 161 women will be randomised per group (two-sided α of 0.05 and 80% power to detect this difference). Allowing for a 10% drop-out rate, the total sample size will be 354 women.

### Outcomes

Primary outcomes of individual trials are summarised in Table 1. Individual trials have different primary outcomes but all trials collect a standard set of outcomes and apply the same definition for each outcome to ensure compatibility for future analysis.

### Independent data monitoring and safety committees

All individual trials have their independent data safety monitoring committees and interim analyses of trial data are planned in some individual trial protocols. Individual trial data monitoring and safety committee charters can be freely accessed [28–32]. An umbrella international data safety monitoring board has been established to provide oversight for all STRIDER trials and will review trial sequential data analysis after the completion of each trial once two trials have been completed.

### Type of analyses

Independent blinded data analysis at the completion of each individual trial will occur on an intention-to-treat basis: that is, for the purpose of analysis, all women will be included in the group to which they have been randomised. Pre-planned subgroup analysis will occur within some individual trials including; assessment of the effect of low placental growth factor (PlGF) at inclusion, umbilical artery Doppler waveform analyses at inclusion (presence or absence of forward flow), and for the effect of other baseline parameters such as gestational age, estimated fetal weight, and participating centre.

### Ancillary and follow-up studies

Each individual trial has ancillary studies underway. These include the effect of sildenafil on: maternal peripheral blood angiogenic factors, myometrial and placental vasculature, maternal haemodynamics, and neonatal cardiac function. Local bio-banking of placental tissue and/or umbilical cord blood is also planned in some trials.

Childhood outcome studies are proposed to assess the important longer-term outcomes of infants born to mothers participating in the STRIDER trials. This will include assessment of neurodevelopmental, cardiovascular, and metabolic outcomes. Assessment at 2–3 years of age is already funded or partially funded in the Netherlands and Ireland. Further funding applications are pending.

### Recruitment status

New Zealand and Australia: Recruitment completed – data analysis in progress.

Canada: Recruiting.

Ireland: Not yet recruiting.

The Netherlands: Recruiting.

United Kingdom: Recruitment completed –primary outcome data submitted for publication.

## Discussion

The STRIDER trials were conceived and designed through international collaboration. The trials are competitively funded at a national level by government funded research agencies within each country. Each individual trial protocol was developed independently by local groups of investigators but all trial designs are similar and all outcomes will be collected in all trials. Each trial will be conducted autonomously, but in close co-operation across the six countries involved. There is a central trial management service hosted at the University of British Columbia, Canada, providing a central randomisation service as well as a central electronic data collection system for each trial. The randomisation service and data collection systems have been designed collaboratively and, although each trial will use its own independent randomisation service and data collection systems these shared resources have reduced overall trial costs and will ensure data compatibility for future analysis in the prospectively planned IPD meta-analyses.

The research teams for all STRIDER trials have regular communication via e-mail, teleconferencing, face-to-face meetings, and a newsletter. Collaboration between the groups is strong with all teams committed to securing funding for long-term infant and childhood outcome studies. These data will contribute to further the systematic review and IPD meta-analysis.

The STRIDER trials are all conducted in order to reduce bias by employing central, stratified randomisation; blinding of all parties through use of matching placebos; central data management focusing on few missing data and dropouts; blinded drawing of conclusions; transparent uploading of IPD data after the trials have been published; no involvement of the pharmaceutical industries selling the product; as well as planning for systematic review of all trials including meta-analyses of individual patient data. These bias eliminating or reducing actions have all been conducted to minimize any risks of systematic errors, that is overestimation of benefits and underestimation of harms [33–40].

The STRIDER trials have all calculated their sample sizes taking into consideration a projected drop-out rate. This gives sample sizes that are inflated according to the assumed risks. We acknowledge this methodology should no longer be undertaken as there is now international consensus to analyse data with multiple imputation [41, 42]. Such analyses will be used for the individual trials, however, we have not amended the sample size calculations as the projected drop-out is small in all trials.

In medicine, a single randomised clinical trial is unlikely to be able to change clinical practice [38]. Therefore, the STRIDER trials have from inception been planned to be systematically reviewed together with any other randomised trials addressing the same topic [22]. Furthermore, in order to better evaluate benefits and harms, the STRIDER trials are planned to be included alone or together with any other trial providing data into individual patient data meta-analyses [22]. The STRIDER Consortium is presently writing up a detailed statistical analysis plan for the systematic review and individual patient data meta-analyses.

The STRIDER Collaboration will ensure the assessment of sildenafil use for the treatment of early onset FGR occurs in a safe and timely manner and should ensure sildenafil is only introduced into clinical practice if reliable data on safety and efficacy supports its use. The extensive IPD data will also provide opportunities to broaden our knowledge of severe early onset FGR, and to explore other applications for sildenafil use if safety and efficacy are established.

## Abbreviations

AC: Abdominal Circumference
CNS: Central Nervous System
EDF: End Diastolic Flow
EFW: Estimate of Fetal Weight
FGR: Fetal Growth Restriction
NICU: Neonatal Intensive Care Unit
PlGF: Placental Growth Factor
